# cMIND Diet, Indoor Air Pollution, and Depression: A Cohort Study Based on the CLHLS from 2011 to 2018

**DOI:** 10.3390/nu15051203

**Published:** 2023-02-27

**Authors:** Ruoyu Wang, Chen Ye, Xiaojie Huang, Mairepaiti Halimulati, Meng Sun, Yuxin Ma, Rui Fan, Zhaofeng Zhang

**Affiliations:** 1 Department of Nutrition and Food Hygiene, School of Public Health, Peking University, Haidian District, Beijing 100191, China; 2 Chinese Center for Disease Control and Prevention, National Institute for Nutrition and Health, Xicheng District, Beijing 100050, China; 3 Beijing’s Key Laboratory of Food Safety Toxicology Research and Evaluation, Haidian District, Beijing 100191, China

**Keywords:** depression, indoor air pollution, cMIND diet, elderly population

## Abstract

This study aims to explore the interaction between a Chinese version of the Mediterranean–DASH intervention for neurodegenerative delay (cMIND) diet and indoor air pollution and its effect on depression among older adults. This cohort study used 2011–2018 data from the Chinese Longitudinal Healthy Longevity Survey. Participants included 2724 adults aged 65 and older without depression. The Chinese version of the Mediterranean–DASH intervention for neurodegenerative delay (cMIND) diet scores ranged from 0 to 12 based on validated food frequency questionnaire responses. Depression was measured using the Phenotypes and eXposures Toolkit. Cox proportional hazards regression models were used to explore the associations, and the analysis was stratified using the cMIND diet scores. A total of 2724 participants (54.3% males and 45.9% 80 years and older) at baseline were included. Living with severe indoor pollution was associated with a 40% increase in the risk of depression (HR: 1.40, 95% CI: 1.07, 1.82) compared to living without indoor pollution. Indoor air pollution exposure was significantly associated with cMIND diet scores. Participants with a lower cMIND diet score (HR: 1.72, 95% CI: 1.24, 2.38) had a greater association with severe pollution than those with a higher cMIND diet score. The cMIND diet may alleviate depression caused by indoor pollution among older adults.

## 1. Introduction

Population aging is accelerating globally at an unprecedented rate; by 2050, people aged 65 and above will comprise approximately 16.7% of the estimated total population of 9.4 billion people [1]. As countries are being affected by COVID-19, the elderly population, as a susceptible population, faces a greater number of major issues, including social isolation and signs of neglect due to ageism, exacerbating the risks of loneliness, anxiety, and depression [2]. Globally, geriatric depression has been a major risk factor for suicide and induces a great economic burden [3], contributing to the global burden of disease [4].

As the country with the highest prevalence of depression worldwide [5], China put forward the mental health action program of Healthy China so that the mental health literacy levels of residents could be boosted to 30% and the depression treatment rate could be increased by 80% by 2030. Remarkably, although China has completed the historic task of eradicating poverty, the distribution of mental health service resources is uneven. An imperceptible reduction in the national prevalence or burden was detected for depression, despite compelling evidence of interventions that reduce its impact. Taken together, these findings highlight the urgent need for developing more effective and widely accepted measures that prevent depression.

In developing countries such as China, urbanization is accompanied by a general switch from solid fuels to clean energy. Notably, accumulating evidence has identified that indoor air pollution may translate into robust targets among all factors associated with depression for prevention [6]. Similarly, the 2019 State of Global Air Report from the nonprofit NGO Health Effects Institute stated that indoor air pollution is an important cause of depression and death, particularly for older women in rural counties [7]. Moreover, the ongoing COVID-19 pandemic has further exacerbated the indoor air pollution problem due to home quarantine. Recent research suggests that indoor pollutants may affect delicate brain structures and functions and have long-lasting adverse effects on depression, especially among elderly individuals [8,9,10,11]. The socioeconomic level of a nation does not drastically change over a short period of time, which means that indoor air pollution has been a long-standing problem.

Recently, emerging evidence has suggested that the relationship between indoor air pollution and human health is likely to be modified by dietary intake. A UK Biobank study of 386,937 participants demonstrated the importance of adherence to a healthy diet in lowering air-pollution-related mortality risk [12]. An interaction between air pollution and plant-based dietary patterns on cognitive function among older adults was also found [13]. Regarding depression, the founding of the International Society for Nutritional Psychiatry Research provides a promising focal point [14]. Recently, the Mediterranean–DASH intervention for neurodegenerative delay (MIND) diet has received considerable attention from the field of psychiatry due to its emphasis on unprocessed foods that are rich in antioxidant nutrients that reduce oxidative stress and inflammation and its ease of adherence [15]. Studies have suggested that the MIND diet may be a promising approach to ward off depression in older adults [16]. However, the MIND diet was based on Western populations, and may not accurately reflect Chinese dietary characteristics. Thus, we previously established for the first time a Chinese version of the MIND (cMIND) diet and verified that the higher cMIND diet score was associated with a lower risk of cognitive impairment [17]. In the hopes of better designing clinical and public health recommendations, conducting good quality research on the cMIND diet in the depression management area has never been more urgent.

Our study aimed to explore the possible interaction between the cMIND diet and indoor air pollution and discover its potential health benefits for depression.

## 2. Materials and Methods

### 2.1. Study Population

This study used 2011–2018 data from the Chinese Longitudinal Healthy Longevity Survey (CLHLS) [18]. The CLHLS is a cohort of a nationally representative sample of Chinese people aged 65 and older in 23 provinces around the country. The Biomedical Ethics Committee of Peking University approved the CLHLS (IRB00001052-13074). A written informed consent form was signed by each participant.

We excluded participants who were under 65 years old, had missing values for indoor air pollution, dietary patterns, and covariates, and had depression at baseline. During the analysis, 2724 participants were included. The inclusion and exclusion criteria of participants are detailed in Figure S1.

### 2.2. Dietary Assessment

The cMIND diet for elderly Chinese individuals was developed according to our previous report [17]. The food groups included whole/refined grains, fresh vegetables, mushrooms/algae, fresh fruits, vegetable/animal oils, fish, soybeans, garlic, nuts, tea, and sugar. Food frequency questionnaires were used to collect dietary data [19,20,21]. We gave a score of 0, 0.5, or 1 for most food items and 0 or 1 for the three items related to staple foods and primary cooking oils. The total score theoretically ranged from 0 to 12, and higher scores indicated better adherence to the cMIND diet. We divided the scores into two halves, including lower or higher cMIND diet scores according to the median level.

### 2.3. Indoor Air Pollution Exposure Assessment

The index for indoor air pollution exposure was created to integrate the impacts of different types of cooking fuels and dampness/mold on indoor air pollution levels. The index was categorized into three grades of pollution: 1 referred to no pollution (clean fuels and no dampness/mold), 2 referred to moderate pollution (polluting fuels or dampness/mold), and 3 referred to severe pollution (polluting fuels and dampness/mold). Coal, wood, and kerosene were considered to be “polluting fuels”, while gas, electricity, and solar energy were considered to be “clean fuels” [22,23]. Dampness/mold indicates fungal contamination indoors [24].

### 2.4. Depression Assessment

We used the consensus measures for the Phenotypes and eXposures (PhenX) Toolkit to measure depression. The items of the PhenX scale were designed using the Composite International Diagnostic Interview Screening Scales (CIDI-SC) and the Diagnostic and Statistical Manual of Mental Disorders (DSM-5) [25]. According to the DSM-5, depression is a major mood disorder that lasts for at least two weeks, with depressed moods and anhedonia as its core features, emphasizing the representation of depression instead of emphasizing the onset of depression. PhenX is a short 2-item self-report screening measure that explores whether the participant has or has not experienced depression symptoms lasting more than two weeks in the past 12 months. We defined depression as being if at least one answer was yes. In prior studies, the PhenX scale was validated and has a reliability coefficient of 0.6766 for the CLHLS [26,27].

### 2.5. Covariates

Based on the behavioral model of late-life depression [28], demographic characteristics, socioeconomic status, health behaviors, and health status were analyzed. The covariates included age (65–79 years or ≥80 years), sex, place of residence (urban or rural), education (formal or informal), financial status (independence or dependence), smoking status (never smoked, former smoker, or current smoker), drinking (never drinking, former drinking, or current drinking), regular exercise (yes or no), and social relationships. Among social relationships, three domains were distinguished: social activities (playing cards/mahjong and participating in organized social activities), social networks (marital status and living alone), and social support (the availability of a person to help when participants had difficulties, when they needed to share some of their thoughts, when they wanted to communicate frequently, and when they were sick) [29]. The indices were scored from 0 to 8, with 0 being no and 1 being yes.

### 2.6. Statistical Analysis

The baseline characteristics of participants were compared by quintiles of diet score using the chi-square test or analysis of variance. An analysis of Cox proportional hazards regression was performed to calculate the hazard ratios (HRs) and 95% confidence intervals (CIs) for the association between indoor air pollution and depression. We included an interaction term in the model to examine whether there was an association between indoor air pollution and cMIND diet scores. Then, the analysis was stratified using the cMIND diet scores and individual food groups, and by combining the cMIND diet and regular exercise. We plotted restricted cubic splines to explore nonlinearity between the cMIND diet scores and depression symptoms. The Wald test was performed to determine whether the relationships were linear or nonlinear.

We conducted an extra sensitivity analysis for the robustness of our main findings. We further applied the competing risk model as a sensitivity analysis because an individual who dies from non-depression-related causes before developing depression cannot develop depression. Additionally, we adjusted the regression models for body mass index (BMI), waist circumference, sleep quality, mini-mental state examination (MMSE) scores, and an index of disease that included five cardiometabolic diseases (hypertension, diabetes, heart disease, cerebrovascular disease, and dyslipidemia) [30]. BMI was classified into four categories: underweight (<18.5 kg/m^2^), normal (18.5–25.0 kg/m^2^), overweight, and obese (≥25.0 kg/m^2^). Waist circumference was categorized dichotomously using a waist circumference of ≥85 cm for men and ≥80 cm for women [31]. Furthermore, we considered the participants who switched from polluting to clean fuels (participants whose primary fuel at baseline had been polluting or clean but changed to another fuel type during follow-up). Finally, we excluded participants with severe stroke, cerebrovascular disease, and cancer. We used R 4.2.0 (R Core Team) for statistical analysis, and two-sided *p* values <0.05 were considered to be significant.

## 3. Results

The baseline characteristics of 2724 CLHLS participants are presented in Table 1. Nearly 45.9% of older adults were 80 years and older. A total of 54.3% were males, 47.6% lived in urban areas, and 55.6% were formally educated. Approximately 22.8% of respondents were current smokers and 20.7% were current drinkers. Based on BMI, 64.8% of participants were of normal weight. A total of 51.5% had central obesity based on waist circumference. Missing data are detailed in Table S1.

**Figure 1:**
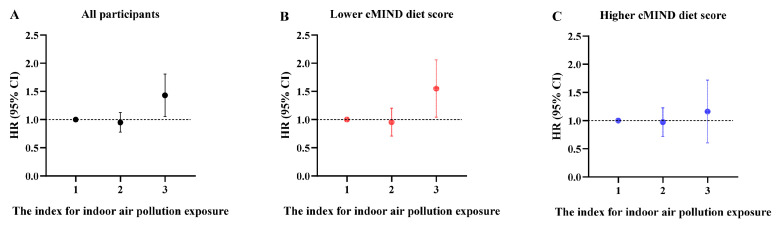
Hazard ratios (95% CI) of incident depression by the index for indoor air pollution exposure, stratified by the cMIND diet score. All participants (**A**), lower cMIND diet score (**B**), and higher cMIND diet score (**C**). No pollution was referred to as 1, moderate pollution was referred to as 2, and severe pollution was referred to as 3.

Table 2 and Figure 1 show the association between indoor air pollution exposure and incident depression. Those living in areas with severe indoor pollution had a 40% increased risk of depression (HR: 1.40, 95% CI: 1.07, 1.82) compared to living in areas without indoor pollution. We found a statistically significant interaction between indoor air pollution exposure and cMIND diet scores, as shown in Table S2. Specifically, compared with groups with a higher cMIND diet score and no pollution, those with a lower cMIND diet score and severe pollution had a higher depression risk (HR: 1.72, 95% CI: 1.24, 2.38). To further unravel these interactions, we stratified the data according to cMIND diet score. We also observed that among participants with lower cMIND diet scores, the corresponding association between severe indoor pollution and depression was much more pronounced (HR: 1.50, 95% CI: 1.07, 2.08) compared to those experiencing no indoor air pollution. Among those with higher cMIND diet scores, there was no difference in the risk of depression with different pollution exposure statuses. Notably, compared to participants engaging in regular exercise, participants not engaging in regular exercise showed an elevated risk of incident depression (HR: 1.25, 95% CI: 1.05, 1.48). To explore the dual protective role of the cMIND diet and exercise on depression, we adjusted the model for the two stratification variables as seen in Table S3. The results showed that indoor pollution had a significant effect on depression only when data from lower cMIND diet scores and irregular exercise were combined. In contrast, indoor pollution was not a risk factor for depression when participants had a higher cMIND diet score or exercised regularly. According to Table 2, participants older than 80 years had a lower risk of depression (HR: 0.82, 95% CI: 0.68, 0.98) than those aged 65 to 79 years. Females had a higher depression risk than males (HR: 1.46, 95% CI: 1.17, 1.81). Furthermore, a reduced risk was observed among urban residents compared to rural residents (HR: 0.82, 95% CI: 0.69, 0.98).

**Figure 2:**
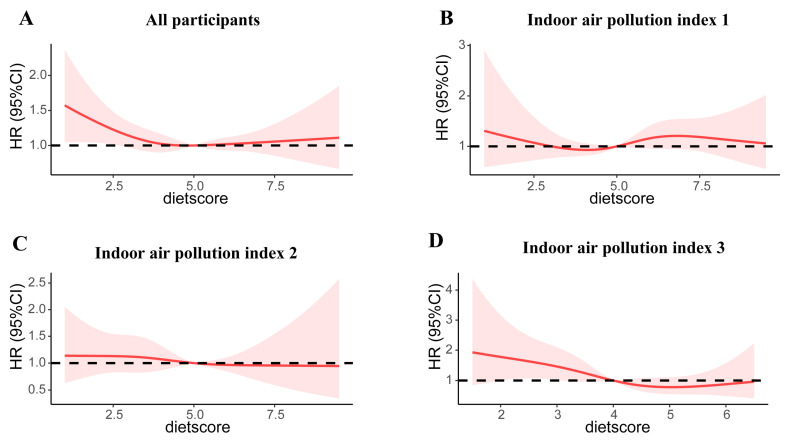
Cubic splines for the cMIND diet score and risks of depression, stratified by the index for indoor air pollution. The shaded area represents 95% CIs. All participants (**A**), indoor air pollution index 1 (**B**), indoor air pollution index 2 (**C**),and indoor air pollution index 3 (**D**).

**Figure 3:**
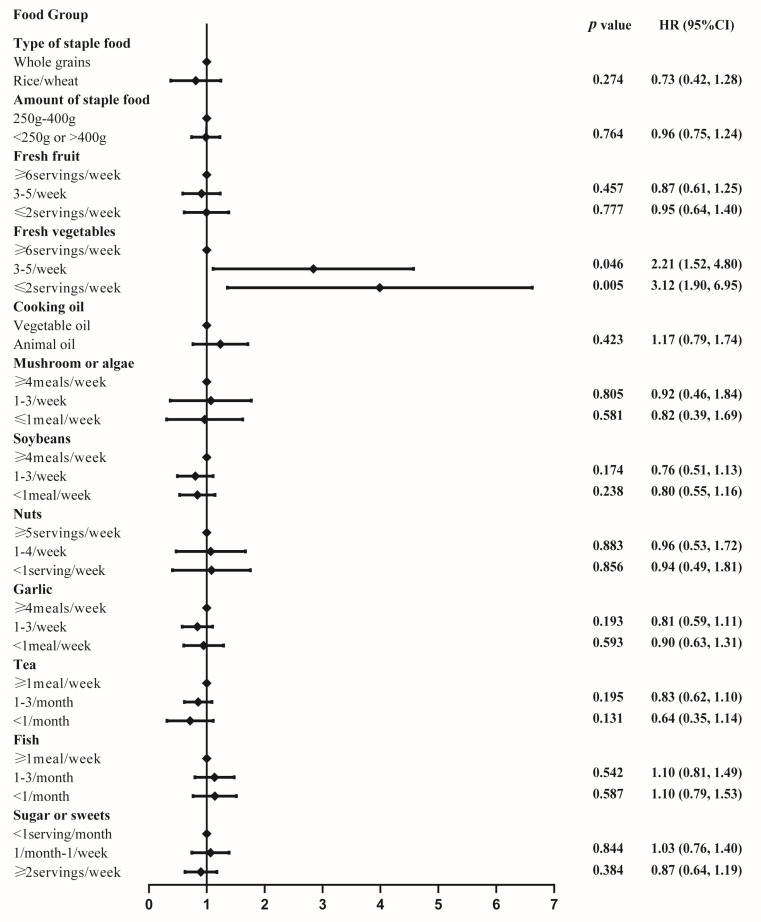
Hazard ratios (95% CI) of incident depression by the index for indoor air pollution, stratified by individual food group.

To further probe a potential nonlinear effect of the cMIND diet on depression stratified by indoor pollution, we used a restricted cubic spline with four knots, as shown in Figure 2. We observed that the test for nonlinearity of the relation between cMIND diet and depression was not significant (*p* = 0.07). As cMIND diet scores were higher than 4, a downward trend in the risk of depression was observed under highly polluted conditions. Furthermore, we probed the risks of depression in indoor air pollution exposure stratified by individual food groups, as seen in Figure 3. The risk estimates of indoor air pollution on depression were lower among participants who consumed fresh vegetables daily.

The results of the main analysis were consistent with those of the sensitivity analysis using competing risk models, as seen in Table S4. This effect of indoor pollution remained after adjusting for extra covariates, as shown in Table S5. Additionally, we found that good sleep quality lowered the risk of depression by 40% compared with poor sleep quality. When cMIND diet scores were higher, the risk of depression did not change regardless of sleep quality. We further investigated how the transition from polluting fuels to clean fuels affected depression, as seen in Table S6. Compared with persistently using polluting fuels, changing cooking fuels from polluting to clean fuels showed a significantly reduced risk of depression (HR: 0.72, 95% CI: 0.56, 0.93). Moreover, the effect of depression risk reduction was observed only when the cMIND diet score was higher rather than lower. For persistent clean fuel users, the risk of depression did not show statistical significance compared with that among previous users of clean fuels. In the sensitivity analyses that excluded those with severe stroke and cerebrovascular disease or those with cancer, higher risks of depression were also observed for severe indoor air pollution, as shown in Table S7.

## 4. Discussion

In this study, we developed an efficient and feasible dietary intervention strategy suitable for Chinese people to prevent depression. Our study showed that living with severe indoor pollution had a 40% increased risk of depression, and the cMIND diet significantly modified the associations of indoor pollution with depression. Our findings could encourage the government to commit to promoting the cMIND diet to achieve the goals of the healthy eating campaign and the mental health action program, as outlined in the blueprint of Healthy China 2030.

To our knowledge, this is the first study to examine the modifying effects of healthy dietary patterns on the relationship between air pollution and depression. Mounting evidence suggests that at least some of the adverse health impacts of indoor pollution are mediated by epigenetic changes that may influence inflammation and depression development [10]. In our study, it was shown that antioxidants and anti-inflammatory nutrients from rich vegetables and fruits [32] and minimally processed foods [33] in the cMIND diet may help to explain how it could mitigate the adverse impacts. Additionally, the cMIND diet developed according to Chinese food culture has the characteristics of strong operability and relatively easy execution because it only follows 10 recommendations and two warnings.

The cMIND diet may not only be beneficial for indoor-air-pollution-associated health outcomes but also has the potential to decrease the socioeconomic burden and inequalities produced by disease and air pollution treatment. In modern scientific history, it has been recognized that indoor air is more important, from a health point of view, than outdoor air [34]. Global lockdowns have also highlighted the impact of domestic sources of pollution. For indoor pollution, we considered dampness/mold, which are topics of concern that have emerged later, differing from previous studies in that they focused on cooking fuels only [35]. Indeed, our findings indicated that dampness/mold may play a certain role in the occurrence of depression. Common problems such as persistent dampness suggest that the burden of unseen indoor air pollution falls disproportionately on the most vulnerable, especially those in poor-quality housing or rural areas. Admittedly, although both domestic and international efforts have been made to reduce indoor air pollution [36,37], it remains a major public health issue, especially in low- and middle-income countries and territories. During this critical window, the cMIND diet, an economical and low-carbon dietary intervention strategy, can be implemented across health care systems and promote health and nutrition equity.

Our findings indicated that the long-term health benefits of regular exercise may mitigate the depression effects associated with exposure to severe indoor air pollution, as well as the positive synergies between the cMIND diet and regular exercise through multiple mechanisms [38,39,40]. Economic lifestyle interventions therefore have important new implications for individuals of lower economic status or who are unable to access good mental health care. In recent years, the incidence of sleep disorders has rapidly increased [41] and they are strongly associated with depression [42]. Our findings underscored that in the case of high cMIND diet compliance, even poor sleep quality did not increase the risk of depression. This suggests that the cMIND diet is a reliable solution for depression induced by poor sleep quality.

Our findings suggested that the switch to clean fuels was promising for the prevention of depression and that the effect was more evident among those with higher cMIND diet scores. This is in agreement with previous studies that focused on switching from polluting to clean fuels [43]. Our present study provides novel evidence in support of the promulgated policy on indoor air pollution in China and sheds new light on combined interventions for depression management, facilitating the implementation of the policy. In our study, the risk of developing depression was stronger among females than males, which was consistent with previous publications [44]. Sex differences may be caused by the differences in biology, activity patterns, or accuracy of measurement [45]. Regarding sociogeographic areas, living in rural areas was associated with a higher risk of depression than living in urban areas because there may be less access to depression treatment in economically underdeveloped areas [46]. Our results helped to identify that elderly individuals in rural areas can be targeted by future policy interventions.

The strengths of our study include the use of a nationally representative sample of older adults covering diverse geographic regions in China, which increased the statistical power to provide a more reliable result. We provided a locally economical, acceptable, and viable pathway for dietary interventions for depression induced by indoor pollution, informing policy interventions to mitigate health inequality in China. Finally, our findings may create a brand new perspective against depression induced by indoor air pollution exposure.

However, our findings should be interpreted with caution. First, self-report questionnaires were used to collect dietary data, so the possibility of recall bias cannot be ruled out. Cooking methods and physical differences in different regions should also be considered for the generalizability of the cMIND diet. Second, no indoor contaminant concentration data were available for our study and indoor pollutant levels were not quantified, but we used the well-accepted determinants of indoor pollution to quantify this variable. Third, we used the PhenX Toolkit rather than clinical diagnoses, which was a source of reporting bias. However, previous research has demonstrated the validity and reliability of the toolkit [26,27]. Finally, as in all observational studies, we were unable to establish causality in our observations, as the mechanisms between diet and depression are complex.

## 5. Conclusions

Our study suggests that greater adherence to the cMIND diet may benefit the prevention of depression induced by indoor air pollution exposure among elderly individuals. The potential for cMIND diet intervention for depression levels of elderly patients is of critical importance. Findings from this and further research could also guide clinicians in recommending dietary intervention for the population. Moreover, a public awareness campaign regarding the health effects of the cMIND diet in elderly individuals should be initiated, followed by practical intervention to reduce mental health problems and promote their quality of life and healthy aging.

## Figures and Tables

**Table 1:** Baseline characteristics of participants based on quintiles of cMIND diet scores with complete data.

Characteristics	Total	Quintile 1	Quintile 2	Quintile 3	Quintile 4	Quintile 5
Range of scores	0–12.00	0–3.50	3.50–4.49	4.50–4.99	5.00–5.99	6.00–12.00
N	2724	504	549	301	604	766
Indoor air pollution exposure *	1.6 (0.6)	1.9 (0.6)	1.8 (0.6)	1.6 (0.6)	1.6 (0.6)	1.4 (0.6)
Age, 65–79 years	1475 (54.1)	227 (45.0)	261 (47.5)	167 (55.5)	347 (57.5)	473 (61.7)
Sex, males	1479 (54.3)	253 (50.2)	255 (46.4)	170 (56.5)	344 (57.0)	457 (59.7)
Urban residence	1296 (47.6)	145 (28.8)	223 (40.6)	140 (46.5)	311 (51.5)	477 (62.3)
With formal education	1515 (55.6)	214 (42.5)	250 (45.5)	158 (52.5)	363 (60.1)	530 (69.2)
Financial independence	1192 (43.8)	154 (30.6)	163 (29.7)	141 (46.8)	279 (46.2)	455 (59.4)
Smoking status						
Never smoked	1661 (61.0)	331 (65.7)	369 (67.2)	189 (62.8)	344 (57.0)	428 (55.9)
Former smoker	442 (16.2)	65 (12.9)	78 (14.2)	39 (13.0)	98 (16.2)	162 (21.1)
Current smoker	621 (22.8)	108 (21.4)	102 (18.6)	73 (24.3)	162 (26.8)	176 (23.0)
Alcohol consumption						
Never drank	1780 (65.3)	359 (71.2)	397 (72.3)	196 (65.1)	380 (62.9)	448 (58.5)
Former drinker	380 (14.0)	61 (12.1)	62 (11.3)	41 (13.6)	90 (14.9)	126 (16.4)
Current drinker	564 (20.7)	84 (16.7)	90 (16.4)	64 (21.3)	134 (22.2)	192 (25.1)
With regular exercise	1230 (45.2)	149 (29.6)	190 (34.6)	111 (36.9)	288 (47.7)	492 (64.2)
Social relationships *	5.7 (1.2)	5.3 (1.2)	5.5 (1.1)	5.6 (1.1)	5.8 (1.1)	6.1 (1.1)
Body mass index						
Underweight	417 (15.3)	128 (25.4)	98 (17.9)	48 (15.9)	80 (13.2)	63 (8.2)
Normal	1764 (64.8)	296 (58.7)	367 (66.8)	188 (62.5)	400 (66.2)	513 (67.0)
Overweight or obese	543 (19.9)	80 (15.9)	84 (15.3)	65 (21.6)	124 (20.5)	190 (24.8)
Waist circumference, normal	1321 (48.5)	303 (60.1)	287 (52.3)	139 (46.2)	290 (48.0)	302 (39.4)
MMSE score *	27.0 (4.2)	25.7 (5.4)	26.6 (4.3)	27.0 (4.2)	27.6 (3.6)	27.8 (3.4)
Sleep quality						
Bad	269 (9.9)	69 (13.7)	64 (11.7)	35 (11.6)	42 (7.0)	59 (7.7)
So so	604 (22.2)	145 (28.8)	131 (23.9)	53 (17.6)	126 (20.9)	149 (19.5)
Good	1851 (68.0)	290 (57.5)	354 (64.5)	213 (70.8)	436 (72.2)	558 (72.8)
Hypertension	974 (35.8)	138 (27.4)	179 (32.6)	121 (40.2)	211 (34.9)	325 (42.4)
Diabetes	354 (13.0)	44 (8.7)	64 (11.7)	45 (15.0)	64 (10.6)	137 (17.9)
Heart diseases	495 (18.2)	73 (14.5)	84 (15.3)	54 (17.9)	105 (17.4)	179 (23.4)
Cerebrovascular disease	344 (12.6)	51 (10.1)	71 (12.9)	39 (13.0)	68 (11.3)	115 (15.0)
Dyslipidemia	245 (9.0)	36 (7.1)	45 (8.2)	21 (7.0)	47 (7.8)	96 (12.5)

Numbers (%) were reported. * Mean (standard deviation) was reported.

**Table 2:** Associations between indoor air pollution exposure and depression.

Variables	All Participants	Lower cMIND Diet Score	Higher cMIND Diet Score
	HR (95% CI)	HR (95% CI)	HR (95% CI)
Indoor air pollution exposure			
No pollution	Ref	Ref	Ref
Moderate pollution	0.94 (0.78, 1.13)	0.93 (0.72, 1.12)	0.95 (0.73, 1.24)
Severe pollution	1.40 (1.07, 1.82) *	1.50 (1.07, 2.08) *	1.07 (0.65, 1.76)
Age, ≥80 years	0.82 (0.68, 0.98) *	0.68 (0.52, 0.90) **	0.94 (0.73, 1.20)
Sex, females	1.46 (1.17, 1.81) **	1.48 (1.08, 2.03) *	1.39 (1.03, 1.88) *
Urban residence	0.82 (0.69, 0.98) *	0.99 (0.76, 1.28)	0.69 (0.54, 0.89) **
With formal education	1.14 (0.94, 1.38)	1.18 (0.90, 1.56)	1.10 (0.84, 1.43)
Financial independence	0.89 (0.74, 1.07)	0.82 (0.62, 1.09)	0.86 (0.67, 1.12)
Smoking status			
Never smoked	Ref	Ref	Ref
Former smoker	1.12 (0.86, 1.46)	1.18 (0.82, 1.70)	1.07 (0.72, 1.59)
Current smoker	1.24 (0.97, 1.57)	1.39 (1.00, 1.95)	1.16 (0.82, 1.63)
Alcohol consumption			
Never drank	Ref	Ref	Ref
Former drinker	1.05 (0.81, 1.37)	1.10 (0.76, 1.59)	1.00 (0.69, 1.45)
Current drinker	0.91 (0.72, 1.14)	0.76 (0.54, 1.07)	1.07 (0.78, 1.47)
Without regular exercise	1.25 (1.05, 1.48) *	0.94 (0.74, 1.20)	1.58 (1.23, 2.02) ***
Social relationships	0.96 (0.89, 1.03)	0.97 (0.86, 1.09)	0.95 (0.86, 1.05)

The regression models were multivariable-adjusted for age (65–79 years or ≥80 years), sex (male or female), residence (urban or rural), education (with or without formal education), financial status (financial independence or dependence), smoking and drinking (never, former, or current smokers/drinkers), regular exercise (yes or no), and social relationships. * *p*  <  0.05, ** *p*  <  0.01, *** *p*  <  0.001.

## Data Availability

The CLHLS questionnaires are available at https://sites.duke.edu/centerforaging/programs/chinese-longitudinal-healthy-longevity-survey-clhls/survey-documentation/questionnaires/. The full datasets used in this analysis are available from the corresponding author upon reasonable request.

## Supplementary Materials

The following supporting information can be downloaded at https://www.mdpi.com/article/10.3390/nu15051203/s1: Figure S1: Flow chart of the included CLHLS participants; Table S1: Baseline characteristics of participants based on quintiles of cMIND diet scores with incomplete data; Table S2: Adjusted hazard ratios for depression based on the joint changes in indoor air pollution exposure and cMIND diet scores; Table S3: The association between indoor air pollution exposure and depression, stratified by cMIND diet scores and exercise; Table S4: The association between indoor air pollution exposure and depression, using the competing risk model; Table S5: The association between indoor air pollution exposure and depression, adjusting for extra covariates; Table S6: The association between switching cooking fuels and depression; Table S7: The association between indoor air pollution exposure and depression after further exclusions.

## References

[1] UN (2019). World Population Ageing.

[2] Monahan C., Macdonald J., Lytle A., Apriceno M., Levy S.R. (2020). COVID-19 and ageism: How positive and negative responses impact older adults and society. Am. Psychol..

[3] Kok R.M., Reynolds C.F. (2017). Management of Depression in Older Adults. JAMA.

[4] Collaborators GMD (2022). Global, regional, and national burden of 12 mental disorders in 204 countries and territories, 1990–2019: A systematic analysis for the Global Burden of Disease Study 2019. Lancet Psychiatry.

[5] People’s Daily (2022). The 2022 National Blue Book of Depression.

[6] Choi K.W., Stein M.B., Nishimi K.M., Ge T., Coleman J.R., Chen C.-Y., Ratanatharathorn A., Zheutlin A.B., Dunn E.C., 23andMe Research Team (2020). An Exposure-Wide and Mendelian Randomization Approach to Identifying Modifiable Factors for the Prevention of Depression. Am. J. Psychiatry.

[7] (2019). State of Global Air Report.

[8] Calderón-Garcidueñas L., Reed W., Maronpot R.R., Henríquez-Roldán C., Delgado-Chavez R., Calderón-Garcidueñas A., Dragustinovis I., Franco-Lira M., Aragón-Flores M., Solt A.C. (2004). Brain Inflammation and Alzheimer’s-Like Pathology in Individuals Exposed to Severe Air Pollution. Toxicol. Pathol..

[9] Calderón-Garcidueñas L., Torres-Jardón R., Avila-Ramírez J., Kulesza R.J., Angiulli A.D. (2015). Air pollution and your brain: What do you need to know right now. Prim. Health Care Res. Dev..

[10] Hunter P. (2020). The health toll of air pollution: Despite global efforts to clean up the air, outdoor and indoor air pollution still have a drastic negative effect on public health. EMBO Rep..

[11] Torres G., Mourad M., Leheste J.R. (2022). Indoor Air Pollution and Decision-Making Behavior: An Interdisciplinary Review. Cureus.

[12] Wang M., Zhou T., Song Q., Ma H., Hu Y., Heianza Y., Qi L. (2022). Ambient air pollution, healthy diet and vegetable intakes, and mortality: A prospective UK Biobank study. Leuk. Res..

[13] Zhu A., Chen H., Shen J., Wang X., Li Z., Zhao A., Shi X., Yan L., Zeng Y., Yuan C. (2022). Interaction between plant-based dietary pattern and air pollution on cognitive function: A prospective cohort analysis of Chinese older adults. Lancet Reg. Health West. Pac..

[14] Sarris J., Logan A.C., Akbaraly T.N., Amminger G.P., Balanzá-Martínez V., Freeman M.P., Hibbeln J., Matsuoka Y., Mischoulon D., Mizoue T. (2015). International Society for Nutritional Psychiatry Research consensus position statement: Nutritional medicine in modern psychiatry. World Psychiatry.

[15] Agarwal P., Wang Y., Buchman A.S., Holland T.M., Bennett D.A., Morris M.C. (2018). MIND Diet Associated with Reduced Incidence and Delayed Progression of Parkinsonism in Old Age. J. Nutr. Health Aging.

[16] Cherian L., Wang Y., Holland T., Agarwal P., Aggarwal N., Morris M.C. (2020). DASH and Mediterranean-Dash Intervention for Neurodegenerative Delay (MIND) Diets Are Associated With Fewer Depressive Symptoms Over Time. J. Gerontol. A Biol. Sci. Med. Sci..

[17] Huang X., Aihemaitijiang S., Ye C., Halimulati M., Wang R., Zhang Z. (2022). Development of the cMIND Diet and Its Association with Cognitive Impairment in Older Chinese People. J. Nutr. Health Aging.

[18] Zeng Y., Dudley L., Poston D., Ashbaugh V., Gu D. (2008). Healthy Longevity in China: Demographic, Socioeconomic, and Psychological Dimensions.

[19] Wong J.E., Parnell W.R., Black K.E., Skidmore P.M. (2012). Reliability and relative validity of a food frequency questionnaire to assess food group intakes in New Zealand adolescents. Nutr. J..

[20] Saeedi P., Skeaff S.A., Wong J.E., Skidmore P.M.L. (2016). Reproducibility and Relative Validity of a Short Food Frequency Questionnaire in 9–10 Year-Old Children. Nutrients.

[21] Ashfield-Watt P., Welch A., Day N., Bingham S. (2004). Is ‘five-a-day’ an effective way of increasing fruit and vegetable intakes?. Public Health Nutr..

[22] Yu K., Qiu G., Chan K.-H., Lam K.-B.H., Kurmi O.P., Bennett D.A., Yu C., Pan A., Lv J., Guo Y. (2018). Association of Solid Fuel Use With Risk of Cardiovascular and All-Cause Mortality in Rural China. JAMA.

[23] Arku R.E., Brauer M., Duong M., Wei L., Hu B., Tse L.A., Mony P.K., Lakshmi P., Pillai R.K., Mohan V. (2020). Adverse health impacts of cooking with kerosene: A multi-country analysis within the Prospective Urban and Rural Epidemiology Study. Environ. Res..

[24] Sofuoglu S.C., Moschandreas D.J. (2003). The link between symptoms of off ice building occupants and in-office air pollution: The Indoor Air Pollution Index. Indoor Air.

[25] Eisinga R., Grotenhuis M.T., Pelzer B. (2013). The reliability of a two-item scale: Pearson, Cronbach, or Spearman-Brown?. Int. J. Public Health.

[26] Hao Z., Ruggiano N., Li Q., Guo Y., Pan X. (2022). Disparities in depression among Chinese older adults with neurodegenerative diseases. Aging Ment. Health.

[27] Su D., Zhang X., He K., Chen Y. (2021). Use of machine learning approach to predict depression in the elderly in China: A longitudinal study. J. Affect. Disord..

[28] Fiske A., Wetherell J.L., Gatz M. (2009). Depression in Older Adults. Annu. Rev. Clin. Psychol..

[29] Kelly M.E., Duff H., Kelly S., Power J.E.M., Brennan S., Lawlor B.A., Loughrey D.G. (2017). The impact of social activities, social networks, social support and social relationships on the cognitive functioning of healthy older adults: A systematic review. Syst. Rev..

[30] Krishnan K.R. (2002). Biological risk factors in late life depression. Biol. Psychiatry.

[31] Yuan Y., Liu K., Zheng M., Chen S., Wang H., Jiang Q., Xiao Y., Zhou L., Liu X., Yu Y. (2022). Analysis of Changes in Weight, Waist Circumference, or Both, and All-Cause Mortality in Chinese Adults. JAMA Netw. Open.

[32] Shishtar E., Rogers G.T., Blumberg J.B., Au R., Jacques P.F. (2020). Long-term dietary flavonoid intake and change in cognitive function in the Framingham Offspring cohort. Public Health Nutr..

[33] Coletro H.N., Mendonça R.D.D., Meireles A.L., Machado-Coelho G.L.L., de Menezes M.C. (2022). Ultra-processed and fresh food consumption and symptoms of anxiety and depression during the COVID—19 pandemic: COVID Inconfidentes. Clin. Nutr. ESPEN.

[34] Sundell J. (2017). Reflections on the history of indoor air science, focusing on the last 50 years. Indoor Air.

[35] Li N., Song Q., Su W., Guo X., Wang H., Liang Q., Liang M., Qu G., Ding X., Zhou X. (2022). Exposure to indoor air pollution from solid fuel and its effect on depression: A systematic review and meta-analysis. Environ. Sci. Pollut. Res. Int..

[36] Abdul-Wahab S.A., En S.C.F., Elkamel A., Ahmadi L., Yetilmezsoy K. (2015). A review of standards and guidelines set by international bodies for the parameters of indoor air quality. Atmos. Pollut. Res..

[37] Committee TUFWDoCC (2022). No. 1 Central Document Xinhua News Agency.

[38] Kandola A., Ashdown-Franks G., Hendrikse J., Sabiston C.M., Stubbs B. (2019). Physical activity and depression: Towards understanding the antidepressant mechanisms of physical activity. Neurosci. Biobehav. Rev..

[39] Weyh C., Krüger K., Strasser B. (2020). Physical Activity and Diet Shape the Immune System during Aging. Nutrients.

[40] Gubert C., Kong G., Renoir T., Hannan A.J. (2020). Exercise, diet and stress as modulators of gut microbiota: Implications for neurodegenerative diseases. Neurobiol. Dis..

[41] Lancet T. (2022). Waking up to the importance of sleep. Lancet.

[42] Yu J., Rawtaer I., Fam J., Jiang M.-J., Feng L., Kua E.H., Mahendran R. (2016). Sleep correlates of depression and anxiety in an elderly Asian population. Psychogeriatrics.

[43] Shao J., Ge T., Liu Y., Zhao Z., Xia Y. (2021). Longitudinal associations between household solid fuel use and depression in middle-aged and older Chinese population: A cohort study. Ecotoxicol. Environ. Saf..

[44] Santomauro D.F., Herrera A.M.M., Shadid J., Zheng P., Ashbaugh C., Pigott D.M., Abbafati C., Adolph C., Amlag J.O., Aravkin A.Y. (2021). Global prevalence and burden of depressive and anxiety disorders in 204 countries and territories in 2020 due to the COVID-19 pandemic. Lancet.

[45] Wenham C., Smith J., Davies S.E., Feng H., Grépin K.A., Harman S., Herten-Crabb A., Morgan R. (2020). Women are most affected by pandemics—Lessons from past outbreaks. Nature.

[46] Thornicroft G., Chatterji S., Evans-Lacko S., Gruber M., Sampson N., Aguilar-Gaxiola S., Al-Hamzawi A., Alonso J., Andrade L., Borges G. (2017). Undertreatment of people with major depressive disorder in 21 countries. Br. J. Psychiatry.

