# Supplementary material for: cMIND Diet, Indoor Air Pollution, and Depression: A Cohort Study Based on the CLHLS from 2011 to 2018
Source: Nutrients. 2023 Feb 27;15(5):1203. doi: 10.3390/nu15051203 (PMC10005708; doi:10.3390/nu15051203)

Supplementary materials

Table S1. Baseline characteristics of participants based on quintiles of cMIND diet scores with incomplete data
Table S2. Adjusted hazard ratios for depression based on the joint changes in indoor air pollution exposure and cMIND diet scores
Table S3. The association between indoor air pollution exposure and depression, stratified by cMIND diet scores and exercise
Table S4. The association between indoor air pollution exposure and depression, using the competing risk model
Table S5. The association between indoor air pollution exposure and depression, adjusting for extra covariates
Table S6. The association between switching cooking fuels and depression
Table S7. The association between indoor air pollution exposure and depression after further exclusions

Figure S1. Flow chart of the included CLHLS participants

Table S1. Baseline characteristics of participants based on quintiles of cMIND diet scores with incomplete data

Characteristics	Total	Quintile 1	Quintile 2	Quintile 3	Quintile 4	Quintile 5
Range of Scores	0-12.00	0-3.50	3.50 - 4.49	4.50 - 4.99	5.00 - 5.99	6.00-12.00
N	5517	1052	1200	679	1243	1343
Indoor air pollution exposure*	1.7 (0.7)	1.9 (0.7)	1.8 (0.7)	1.6 (0.6)	1.6 (0.6)	1.4 (0.6)
Age, 65–79 years	1803 (31.4)	240 (22.8)	311 (25.9)	214 (31.5)	425 (34.2)	613 (45.6)
Sex, males	2513 (45.6)	412 (39.2)	483 (40.3)	293 (43.2)	591 (47.5)	734 (54.7)
Urban residence	2615 (47.4)	357 (33.9)	726 (60.5)	294 (43.3)	614 (49.4)	876 (65.2)
With formal education	2201 (39.9)	262 (24.9)	377 (31.4)	240 (35.3)	553 (44.5)	769 (57.3)
Financial independence	1629 (29.5)	178 (16.9)	249 (20.8)	182 (26.8)	389 (31.3)	631 (47.0)
Smoking status						
Never smoker	3603 (65.3)	705 (67.0)	830 (69.2)	480 (70.7)	776 (62.4)	812 (60.5)
Former smoker	867 (15.7)	141 (13.4)	174 (14.5)	92 (13.5)	207 (16.7)	253 (18.8)
Current smoker	957 (17.3)	142 (13.5)	185 (15.4)	104 (15.3)	255 (20.5)	271 (20.2)
Alcohol consumption						
Never drinker	3749 (68.0)	747 (71.0)	875 (72.9)	491 (72.3)	809 (65.1)	827 (61.6)
Former drinker	714 (12.9)	126 (12.0)	132 (11.0)	76 (11.2)	179 (14.4)	201 (15.0)
Current drinker	932 (16.9)	112 (10.6)	174 (14.5)	109 (16.1)	243 (19.5)	294 (21.9)
With regular exercise	1622 (29.4)	162 (15.4)	244 (20.3)	158 (23.3)	377 (30.3)	681 (50.7)
Social relationships*	5.2 (1.4)	4.6 (1.7)	5.1 (1.2)	5.2 (1.2)	5.3 (1.2)	5.7 (1.2)
Body mass index						
Underweight	1127 (20.4)	265 (25.2)	273 (22.8)	146 (21.5)	269 (21.6)	174 (13.0)
Normal	3085 (55.9)	536 (51.0)	675 (56.3)	390 (57.4)	694 (55.8)	790 (58.8)
Overweight or obese	856 (15.5)	124 (11.8)	135 (11.3)	91 (13.4)	195 (15.7)	311 (23.2)
Waist circumference, normal	2830 (51.3)	613 (58.3)	658 (54.8)	355 (52.3)	656 (52.8)	548 (40.8)
MMSE score*	21.6 (10.0)	17.0 (11.3)	20.2 (10.2)	21.6 (9.7)	23.1 (9.0)	25.0 (7.9)
Sleep quality						
Bad	499 (9.0)	142 (13.5)	107 (8.9)	56 (8.2)	96 (7.7)	98 (7.3)
So so	1334 (24.2)	309 (29.4)	315 (26.3)	160 (23.6)	280 (22.5)	270 (20.1)
Good	3684 (66.8)	601 (57.1)	778 (64.8)	463 (68.2)	867 (69.8)	975 (72.6)
Hypertension	1984 (36.0)	332 (31.6)	408 (34.0)	254 (37.4)	465 (37.4)	525 (39.1)
Diabetes	738 (13.4)	100 (9.5)	153 (12.8)	92 (13.5)	167 (13.4)	226 (16.8)
Heart diseases	1089 (19.7)	167 (15.9)	224 (18.7)	109 (16.1)	256 (20.6)	333 (24.8)
Cerebrovascular disease	824 (14.9)	146 (13.9)	181 (15.1)	98 (14.4)	176 (14.2)	223 (16.6)
Dyslipidemia	501 (9.1)	65 (6.2)	108 (9.0)	49 (7.2)	95 (7.6)	184 (13.7)

Number (%) were reported.

*Mean (standard deviation) was reported.

Table S2. Adjusted hazard ratios for depression based on the joint changes in indoor air pollution exposure and cMIND diet scores

Variables	HR (95% CI)
Higher cMIND diet score and no pollution	Ref
Higher cMIND diet score and moderate pollution	1.04 (0.81, 1.34)
Higher cMIND diet score and severe pollution	1.16 (0.71, 1.89)
Lower cMIND diet score and no pollution	1.20 (0.94, 1.54)
Lower cMIND diet score and moderate pollution	1.03 (0.79, 1.36)
Lower cMIND diet score and severe pollution	1.72 (1.24, 2.38)**

The regression models were multivariable-adjusted for age (65–79 years or ≥80 years), sex (male or female), residence (urban or rural), education (with or without formal education), financial status (financial independence or dependence), smoking and drinking (never, former, or current smokers/drinkers), regular exercise (yes or no), and social relationships.

**$P < 0.01$.

Table S3. The association between indoor air pollution exposure and depression, stratified by cMIND diet scores and exercise

Variables	HR (95% CI)
Higher cMIND diet score and regular exercise	
Indoor air pollution exposure	
No pollution	Ref
Moderate pollution	0.93 (0.63, 1.37)
Severe pollution	0.76 (0.32, 1.79)
Higher cMIND diet score and irregular exercise	
Indoor air pollution exposure	
No pollution	Ref
Moderate pollution	0.86 (0.62, 1.19)
Severe pollution	1.31 (0.87, 1.98)
Lower cMIND diet score and regular exercise	
Indoor air pollution exposure	
No pollution	Ref
Moderate pollution	0.94 (0.65, 1.36)
Severe pollution	1.37 (0.74, 2.55)
Lower cMIND diet score and irregular exercise	
Indoor air pollution exposure	
No pollution	Ref
Moderate pollution	1.03 (0.66, 1.60)
Severe pollution	2.09 (1.18, 3.71)*

The regression models were multivariable-adjusted for age (65–79 years or ≥80 years), sex (male or female), residence (urban or rural), education (with or without formal education), financial status (financial independence or dependence), smoking and drinking (never, former, or current smokers/drinkers), and social relationships.

*$P < 0.05$.

Table S4. The association between indoor air pollution exposure and depression, using the competing risk model

Variables	HR (95% CI)
All participants	
Indoor air pollution exposure	
No pollution	Ref
Moderate pollution	0.87 (0.63, 1.21)
Severe pollution	1.74 (1.11, 2.72)*
Stratified by cMIND diet score	
Lower cMIND diet score	
Indoor air pollution exposure	
No pollution	Ref
Moderate pollution	0.87 (0.67, 1.13)
Severe pollution	1.65 (1.13, 2.42)**
Higher cMIND diet score	
Indoor air pollution exposure	
No pollution	Ref
Moderate pollution	0.85 (0.58, 1.24)
Severe pollution	1.14 (0.58, 2.24)
Stratified by cMIND diet score and exercise	
Higher cMIND diet score and regular exercise	
Indoor air pollution exposure	
No pollution	Ref
Moderate pollution	0.77 (0.44, 1.33)
Severe pollution	0.87 (0.44, 1.74)
Higher cMIND diet score and irregular exercise	
Indoor air pollution exposure	
No pollution	Ref
Moderate pollution	0.73 (0.49, 1.07)
Severe pollution	1.37 (0.74, 2.56)
Lower cMIND diet score and regular exercise	
Indoor air pollution exposure	
No pollution	Ref
Moderate pollution	0.80 (0.49, 1.32)
Severe pollution	0.90 (0.56, 1.44)
Lower cMIND diet score and irregular exercise	
Indoor air pollution exposure	
No pollution	Ref

Moderate pollution	1.18 (0.82, 1.71)
Severe pollution	1.13 (0.70, 1.83)

The regression models were multivariable-adjusted for age (65–79 years or ≥80 years), sex (male or female), residence (urban or rural), education (with or without formal education), financial status (financial independence or dependence), smoking and drinking (never, former, or current smokers/drinkers), and social relationships.

*$P < 0.05$, **$P < 0.01$.

Table S5. The association between indoor air pollution exposure and depression, adjusting for extra covariates

Variables	All participants	Lower cMIND diet score	Higher cMIND diet score
	HR (95% CI)	HR (95% CI)	HR (95% CI)
Indoor air pollution exposure			
No pollution	Ref	Ref	Ref
Moderate pollution	1.08 (0.82, 1.44)	0.93 (0.59, 1.46)	1.20 (0.83, 1.74)
Severe pollution	1.86 (1.24, 2.79)**	2.32 (1.35, 3.99)**	1.18 (0.59, 2.37)
Age, ≥80 years	1.04 (0.79, 1.36)	0.94 (0.65, 1.37)	1.19 (0.79, 1.80)
Sex, females	1.62 (1.16, 2.28)**	1.88 (1.21, 2.91)**	1.32 (0.77, 2.27)
Urban residence	1.00 (0.76, 1.31)	1.05 (0.72, 1.52)	0.92 (0.61, 1.39)
With formal education	1.18 (0.89, 1.57)	1.16 (0.79, 1.69)	1.27 (0.82, 1.95)
Financial independence	0.94 (0.71, 1.26)	0.80 (0.54, 1.18)	1.06 (0.68, 1.66)
Smoking status			
Never smoker	Ref	Ref	Ref
Former smoker	1.18 (0.81, 1.73)	1.52 (0.92, 2.50)	0.86 (0.45, 1.64)
Current smoker	1.12 (0.77, 1.61)	1.44 (0.90, 2.30)	0.76 (0.42, 1.38)
Alcohol consumption			
Never drinker	Ref	Ref	Ref
Former drinker	1.02 (0.70, 1.49)	1.08 (0.65, 1.79)	0.83 (0.45, 1.53)
Current drinker	0.92 (0.65, 1.29)	0.80 (0.50, 1.30)	1.15 (0.68, 1.96)
Without regular exercise	1.12 (0.86, 1.45)	1.04 (0.73, 1.47)	1.21 (0.80, 1.82)
Social relationships	0.99 (0.88, 1.11)	1.04 (0.88, 1.21)	0.94 (0.79, 1.12)
Body mass index			
Underweight	Ref	Ref	Ref
Normal	1.04 (0.72, 1.48)	1.11 (0.63, 1.95)	0.98 (0.60, 1.60)
Overweight or obese	1.19 (0.76, 1.84)	1.27 (0.64, 2.52)	1.08 (0.60, 1.94)
Central obesity	0.76 (0.58, 1.00)*	0.77 (0.53, 1.12)	0.74 (0.49, 1.14)
MMSE score	1.04 (1.00, 1.08)	1.06 (1.00, 1.13)*	1.01 (0.96, 1.06)
Sleep quality			
Bad	Ref	Ref	Ref
So so	0.76 (0.49, 1.17)	0.58 (0.32, 1.06)	0.87 (0.46, 1.65)
Good	0.60 (0.41, 0.87)**	0.43 (0.26, 0.73)**	0.81 (0.46, 1.43)
Index of disease	1.04 (0.96, 1.12)	1.00 (0.89, 1.11)	1.05 (0.93, 1.18)

The regression models were multivariable-adjusted for age (65–79 years or ≥80 years), sex (male or female), residence (urban or rural), education (with or without formal education), financial status (financial independence or dependence), smoking and drinking (never, former, or current smokers/drinkers), regular exercise (yes or no) social relationships, body mass index (BMI), waist circumference, sleep quality, the MMSE scores, and index of disease including five cardiometabolic diseases.

**P* < 0.05, ***P* < 0.01.

Table S6. The association between switching cooking fuels and depression

Variables	All participants	Lower cMIND diet score	Higher cMIND diet score
	HR (95% CI)	HR (95% CI)	HR (95% CI)
Switch from polluting fuels to clean fuels			
Always polluting fuels	Ref	Ref	Ref
Polluting to clean fuels	0.72 (0.56, 0.93)*	0.80 (0.59, 1.10)	0.63 (0.41, 0.96)*
Switch from clean fuels to polluting fuels			
Alwaysclea fuels	Ref	Ref	Ref
Clean to polluting fuels	1.22 (0.87, 1.71)	1.42 (0.88, 2.29)	0.97 (0.60, 1.58)

The regression models were multivariable-adjusted for age (65–79 years or ≥80 years), sex (male or female), residence (urban or rural), education (with or without formal education), financial status (financial independence or dependence), smoking and drinking (never, former, or current smokers/drinkers), regular exercise (yes or no), and social relationships.

*$P < 0.05$.

Table S7. The association between indoor air pollution exposure and depression after further exclusions

Variables	All participants	Lower cMIND diet score	Higher cMIND diet score
	HR (95% CI)	HR (95% CI)	HR (95% CI)
Excluding severe stroke and cerebrovascular disease			
Indoor air pollution exposure			
No pollution	Ref	Ref	Ref
Moderate pollution	0.88 (0.70, 1.01)	0.99 (0.72, 1.34)	0.78 (0.54, 1.12)
Severe pollution	1.32 (1.05, 1.83)*	1.53 (1.02, 2.30)*	1.00 (0.52, 1.91)
Excluding severe cancer			
Indoor air pollution exposure			
No pollution	Ref	Ref	Ref
Moderate pollution	0.88 (0.70, 1.11)	0.98 (0.72, 1.33)	0.76 (0.53, 1.11)
Severe pollution	1.31 (1.05, 1.83)*	1.47 (1.08, 2.21)*	1.12 (0.58, 2.16)

The regression models were multivariable-adjusted for age (65–79 years or ≥80 years), sex (male or female), residence (urban or rural), education (with or without formal education), financial status (financial independence or dependence), smoking and drinking (never, former, or current smokers/drinkers), regular exercise (yes or no), and social relationships.

*$P < 0.05$.

Figure S1. Flow chart of the included CLHLS participants

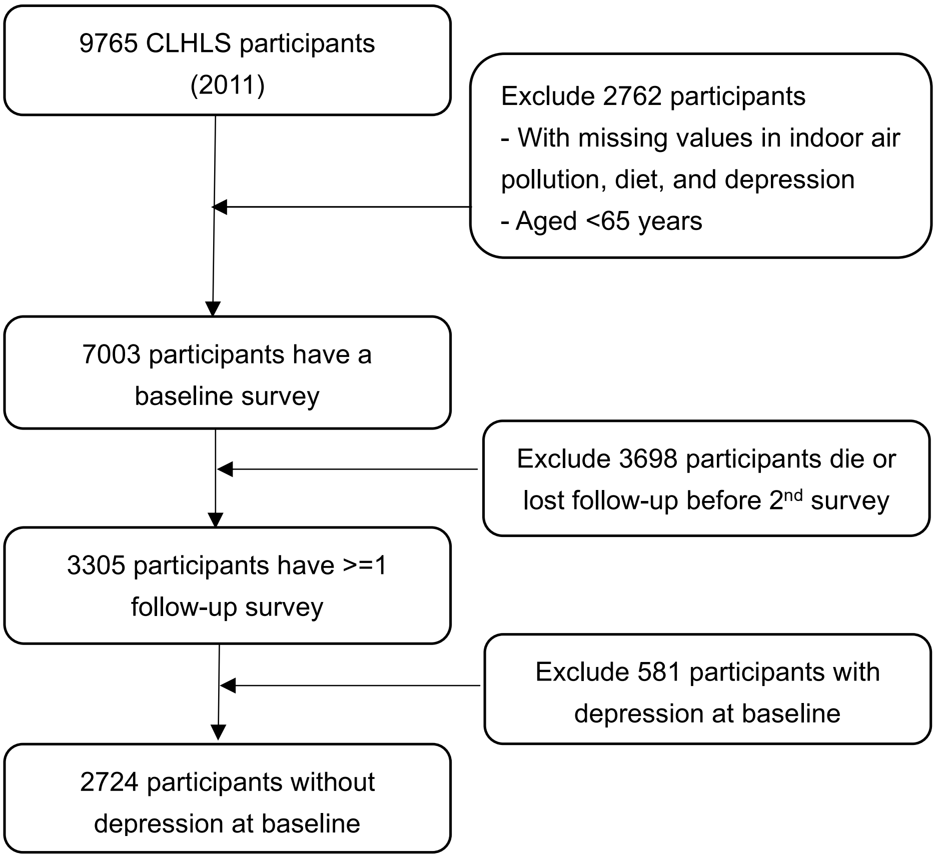

Supplement: Supplementary file 1 [file nutrients-15-01203-s001.zip › supplementary materials.pdf]
